# Newcastle Disease Virus in Madagascar: Identification of an Original Genotype Possibly Deriving from a Died Out Ancestor of Genotype IV

**DOI:** 10.1371/journal.pone.0013987

**Published:** 2010-11-15

**Authors:** Olivier F. Maminiaina, Patricia Gil, François-Xavier Briand, Emmanuel Albina, Djénéba Keita, Harentsoaniaina Rasamoelina Andriamanivo, Véronique Chevalier, Renaud Lancelot, Dominique Martinez, R. Rakotondravao, Jean-Joseph Rajaonarison, M. Koko, Abel A. Andriantsimahavandy, Véronique Jestin, Renata Servan de Almeida

**Affiliations:** 1 FOFIFA-DRZV, Antananarivo, Madagascar; 2 CIRAD, BIOS Department, UMR CMAEE, Montpellier, France; 3 Anses-Ploufragan Plouzané Laboratory, VIPAC Unit, Ploufragan, France; 4 CIRAD, ES Department, UPR AGIRS, Montpellier, France; 5 Antananarivo University Madagascar, Antananarivo, Madagascar; Veterinary Laboratories Agency, United Kingdom

## Abstract

In Madagascar, Newcastle disease (ND) has become enzootic after the first documented epizootics in 1946, with recurrent annual outbreaks causing mortality up to 40%. Four ND viruses recently isolated in Madagascar were genotypically and pathotypically characterised. By phylogenetic inference based on the F and HN genes, and also full-genome sequence analyses, the NDV Malagasy isolates form a cluster distant enough to constitute a new genotype hereby proposed as genotype XI. This new genotype is presumably deriving from an ancestor close to genotype IV introduced in the island probably more than 50 years ago. Our data show also that all the previously described neutralising epitopes are conserved between Malagasy and vaccine strains. However, the potential implication in vaccination failures of specific amino acid substitutions predominantly found on surface-exposed epitopes of F and HN proteins is discussed.

## Introduction

Newcastle disease (ND) is a highly contagious and widespread disease which causes severe economic losses in domestic poultry, especially in chickens [1], [2]. In Africa, ND is the major constraint to rural chicken development. This disease is listed as a notifiable disease by the World Organisation for Animal Health (OIE) [3]. The causative agent of the disease is Newcastle disease virus (NDV), also designated avian paramyxovirus serotype 1 (APMV-1), which belongs to the genus *Avulavirus* within the family *Paramyxoviridae* [4], [5]. The genome comprises a single stranded negative sense RNA that encodes the RNA-dependent RNA polymerase (L gene), the haemagglutinin-neuraminidase (HN gene), the fusion (F gene) and matrix (M gene) proteins, the phosphoprotein (P gene) and the nucleoprotein (NP gene). The genome is predicted to be 15186, 15192 or 15198 nucleotides (nt) in length, always a multiple of six nt, which fulfils the so-called ‘*rule of six*’ for optimised replication [6], [7], [8], [9], [10]. Based on the analysis of nucleotide sequence of the F protein gene, 10 different genotypes (I–X) or 6 different lineages (1–6) of NDV have been identified so far. The genotypes VI (lineage 4) and VII (lineage 5) are further divided into eight (a–h) and five (a–e) subgenotypes/sublineages, respectively [11], [12], [13], [14], [15], [16], [17], [18], [19]. APMV-1 strains can be categorised according to their virulence into highly (velogenic), intermediate (mesogenic) or non virulent (lentogenic). The pathogenicity of APMV-1 isolates is assessed on the basis of *in vivo* tests including the intracerebral pathogenicity index (ICPI) in 1-day-old chickens, the mean time of death (MDT) of embryonated specific-pathogen-free hen's eggs after inoculation and the intravenous pathogenicity index (IVPI) in 6-week-old chickens [3]. The molecular basis for these different levels of pathogenicity is known to be linked to the sequence of cleavage site of the precursor fusion protein (F_0_). At this position, a pathogenic NDV strain (velogenic and mesogenic) has at least one extra pair of basic amino acids motif ^112^X-R-X-R/K-R-F^117^ [20] and can be cleaved by a wide range of proteases of the furin family in different host cells [21].

Since ND was first described in 1926, three worldwide panzootics have occurred [20]. The first panzootic (1926 to 1960) was caused by viruses belonging to genotypes II–III–IV and the second (1960 to 1973) and third (1970–1980) ones by genotypes V–VI. Moreover, severe outbreaks in Western and Southern Europe [22], [23], South Africa [24] and Taiwan [25] in the 90's were caused by genotype VII, the currently circulating genotype in Asia, Africa and Europe. In Madagascar, ND was firstly described in 1946 [26] and since then, outbreaks were regularly reported on the whole island mainly in the rural poultry sector [27]. While the vaccination rate is estimated to reach 100% in commercial farms in Madagascar, probably less than 10% of the free-range poultries are duly vaccinated. ND is considered to induce more than 40% of mortality in such non protected poultries [28]. In spite of the importance and endemicity of ND in Madagascar, no data is available about the virus variants involved in clinical cases and/or maintenance of this disease in the island. In this study, four APMV-1 strains from Madagascar (named here MG group) were isolated in 1992 and 2008 and were molecularly characterised. Two of these strains were fully sequenced while the two other ones had only F and HN genes sequenced. Phylogenetic analyses showed that the MG group strains clustered within a new distinct genotype, closer to old genotypes. This study represents the first molecular characterisation of APMV-1 circulating in Madagascar and provides evidence on the existence of a new genotype close to an old died out genotype.

## Materials and Methods

### Ethics statement

All animal experiments (ICPI tests) were conducted according to internationally approved OIE standards, under authorizations set forth by the director of the veterinary services of Côtes d'Armor on behalf of the Prefect of Côtes d'Armor (N° 22-18) and the Director of the veterinary services of Hérault on behalf of the Prefect of Hérault (N° 34-114). Certificates of authorization are available from the authors upon request.

### Origin and isolation of virus strains

Synthetic information about the Madagascar isolates is provided in Table 1. An “old” strain (MG-1992) was first isolated by the *Centre National de Recherche Appliquée au Développement Rural* (FOFIFA-DRZV) in 1992, after suspicion of avian influenza or ND in a dead fowl assumed to be vaccinated against ND (La Sota vaccine). The three other isolates were collected in Madagascar during a surveillance programme in African wetlands led in 2007 and 2008 by the *Centre de Cooperation Internationale en Recherche Agronomique pour le Développement* (CIRAD), Montpellier, France and the FOFIFA-DRZV, Antananarivo, Madagascar. The MG-725/08 strain was recovered both from cloacal and tracheal swabs from an unvaccinated apparently healthy chicken. Another strain (MG39-04/08) was isolated from a dead chicken in a commercial farm: this chicken was initially vaccinated with the HB1 strain and boosted with the La Sota vaccine. The last isolate (MG-Meola/08) was recovered from a non vaccinated backyard poultry.

**Table 1:** NDV isolates from Madagascar used in this study.

Strain	Code	Region/Country	Statut		Sequence	
			Clinic	Vaccine	size (nt)	Covering full genes
APMV1/Chicken/MG/1992	MG-1992	Ivato-Madagascar	Dead laying chicken	La Sota	15082	NP, P, M, F, HN and L
APMV1/Chicken/MG/725/2008	MG-725/08	Mahitsy-Madagascar	Healthy rural poultry	NV^a^	15097	NP, P, M, F, HN and L
APMV1/Chicken/MG/39-4/2008	MG-39-04/08	Ivato-Madagascar	Dead broiler chicken	La Sota	4249	F and HN
APMV1/Chicken/MG/Meola/2008	MG-Meola/08	Tsarahonenana-Madagascar	Sick grower fighter cock	NV	3966	F and HN

a : not vaccinated.

Samples of brain tissues or cloacal/tracheal swabs positives in the APMV-1 specific PCR (protocol based on M gene recommended by the reference laboratory of the OIE) were further processed for virus isolation by inoculation into 9-day old chicken embryonated eggs. All isolates were grown in less than 3 passages on eggs and tested by PCR before molecular sequencing.

### In vivo assay

The intracerebral pathogenicity index (ICPI) was determined according to international OIE standards (OIE, 2009) for two isolates of the MG group (MG-1992 and MG-725/08). Briefly, fresh allantoic fluid with a HA titre >1/16 was diluted 10^−1^ in sterile isotonic saline buffer. Following filtration through 0.22 or 0.45 µm filters this diluted virus was injected intracerebraly (0.05 ml) into 10 one day-old chick hatched from embryonated SPF hens' eggs. Whereas each inoculum was checked for lack of bacterial and fungal contamination following culture on trypto casein soja and sabouraud media, the birds were examined at 24h intervals for 8 days and given a score (0, 1 or 2) according to their status (respectively healthy, sick or dead). The ICPI was calculated as the total of individual scores during 8 days divided by 80 (the number of days x the number of chickens).

### RNA isolation, cDNA synthesis and nucleotide sequencing

For reverse transcription and PCR reactions, RNA was extracted from allantoic fluids by using the Nucleospin® RNA Virus kit (MachereyNagel) following the manufacturer's instructions. The cDNA transcriptions were carried out by using the First-strand cDNA synthesis kit (GE Healthcare). Six pairs of oligonucleotide primers previously published [13], [29] or designed in this study (Table S1 in supporting material), using BioEdit version 7.0.9.0 and Vector NTI software version 11.0 (©2008 Invitrogen Corporation), were used to amplify six overlapping DNA fragments to generate the complete sequences of F and HN protein genes. All DNA fragments were sequenced in both directions by Cogenics Genome Express S.A. (Meylan-France). For the rest of the genome, 23 overlapping PCR were realized with Platinum Taq DNA polymerase (Platinum® Taq DNA Polymerase High Fidelity, Invitrogen). The DNA sequences were determined in both senses using the Big dye Terminator v3.1 cycle sequencing kit (Applied Biosystems) according to the manufacturer's instructions and using the same primers as to PCR reactions.

### Alignment of the F and HN predicted amino acid sequences and phylogenetic analyses

The sequences of the four isolates were compared with previously reported NDV sequences representative of different genotypes available in GenBank. A 374 nucleotide (nt) fragment of a variable portion of the F protein gene (47 to 421), including the F_0_ cleavage site and a 1713 nt (92 to 1805) of HN gene were processed by Clustal W [30] in alignX program included into Vector NTI software suite (version 11.0). The nucleotide sequence databank accession numbers of ND viruses used in this study are shown in supporting Table S2 of supporting material. Alignment of complete amino acid sequences of F and HN was processed by alignX program in Vector NTI software and finalised using MEGA4 software (version 4.1). Phylogenetic relationships and nucleotide divergence analyse (intra and inter genotype) were established with MEGA4 [31] using the Kimura 2-parameter correction in which the transition/transversion ratio was estimated from the data [32]. The statistical significance of the tree topology generated with the neighbor-joining algorithm was evaluated by 1,000 bootstrap resamplings of the data [33]. To confirm the robustness of the genetic groupings obtained by the phylogenetic analysis on the partial fragment (374 nt) a phylogenetic tree based on full-genome sequences of two isolates of the MG group (MG-1992 and MG-725/08) and 86 other full-genome sequences belonging to different genotypes were further carried out using the same parameters used for F and HN analyses. Putative recombination events in the two full-genomes of MG isolates were examinated using the recombination detection program RDP 3.0b [34].

## Results

### Determination of *in vivo* pathogenicity (ICPI test)

ICPI values were determined for the isolates MG-1992 and MG-725/08. These isolates showed a very high ICPI of 1.9 being classified into a velogen-type NDV (Table 2).

**Table 2:** F gene referential NDV strains used in this study.

Strain		Class or Genogroup	ICPI^a^ value	Cleavage site							
Identification	Accession number			←---------------------------------- F_2_→					*	←F_1_-------→	
				112	113	114	115	116		117	118
				R	R	Q	R	R	*	F	I
DE R49/99	DQ097393	Cl I (6)	nd	G	-	-	G	-	*	L	V
GO1US DCKI	AY626266	Cl I (6)	nd	E	-	-	E	-	*	L	V
Herts33/56 (PEI)	AY170140	H33 (w)^b^	nd	-	-	-	-	-	*	-	-
Herts33 (L)	AY170138	H33 (w)^c^	nd	-	-	-	-	-	*	-	-
Ulster/67	AY562991	I (1)	0,0	G	K	-	G	-	*	L	-
Ethiopie Panvac (2/P2)	AY175720	I (1)	0,0	G	K	-	G	-	*	L	-
PHY-LMV42/66	DQ097394	I (1)	0,0	G	K	-	G	-	*	L	-
Hitchner B1	AF309418	II (2)	0,2–0,5	G	-	-	G	-	*	L	-
La Sota	AF077761	II (2)	0,2–0,5	G	-	-	G	-	*	L	-
Zimbabwe AV862/95	AY175710	II (2)	nd	G	-	-	G	-	*	L	-
Zambie AV 72/95	AY175708	II (2)	nd	G	-	-	G	-	*	L	-
Mukteswar	EF201805	III (3a)	1,4	-	-	-	-	-	*	-	-
Guangxi5/2000	DQ485259	III (3a)	nd	-	-	-	-	-	*	-	-
Herts/33 (de Leeuw)	AY741404	IVea (3b)	1,99	-	-	-	-	-	*	-	-
BG 60–81	AF402129	IVea (3b)	nd	-	-	-	-	-	*	-	-
BG 5–67	AF402104	IVbg (3b)	nd								
SIMF/64	AJ243390	IVbg (3b)	nd	-	-	-	-	-	*	-	-
Soudan 72 AV 2203	AY135753	IVit (3b)	nd	-	-	-	-	-	*	-	-
DE-191/77	AF525378	IVit (3b)	nd	-	-	-	-	-	*	-	-
IT-48/68	AF297969	IVit (3b)	nd	-	-	-	-	-	*	-	-
MA-307/77^c^	EU604259	IV (3b)	nd	-	-	-	-	-	*	-	V
MA-13/02^c^	DQ096598	IV (3b)	nd	-	-	-	-	-	*	-	V
Tanzania AV 1300/95	AY175687	V (3c)	nd	-	-	-	K	-	*	-	V
Mexico468/01	EU518685	V (3c)	nd	-	-	-	K	-	*	-	V
Brasil AV1769/90	AY175649	V (3c)	nd	-	-	-	K	-	*	-	V
HR-111/01	AY150162	VI (4)	nd	K	-	-	K	-	*	-	-
Soudan SD-4/75	AY151384	VI (4)	nd	-	-	-	K	-		-	-
Egypte EG-3/87	AY150111	VI (4)	nd	-	-	-	K	-		-	-
DE 61/93	AY150135	VI (4)	nd	-	-	K	K	-	*	-	-
Strain NA	DQ659677	VII (5)	nd	-	-	-	K	-	*	-	-
MZ 13/94	AF136775	VII (5)	nd	-	-	-	K	-	*	-	-
Botswana ZA148/UP/98	AY210507	VII (5)	nd	-	-	-	K	-	*	-	-
South Africa ZA606/UP/00	AY210497	VIII (3d)	nd	-	-	-	K	-	*	-	-
Singapore SG-4H/65	AF136786	VIII (3d)	nd	-	-	-	K	-	*	-	V
F48E9	AY508514	IX (3e)	1,89	-	-	-	-	-	*	-	-
SBD02	DQ227252	IX (3e)	nd	-	-	-	-	-	*	-	-
TJ03	DQ227244	IX (3e)	nd	-	-	-	-	-	*	-	-
TW/69	AF083959	X (3f)	nd	-	-	-	K	-	*	-	-
TW/95-3	AF083970	X (3f)	1,68	-	-	-	K	-	*	-	-
**MG-1992**	HQ266603	XI (3g)	1,9	-	-	R	-	-	*	-	V
**MG-725/2008**	HQ266602	XI (3g)	1,9	-	-	R	-	-	*	-	V
**MG-39-4/2008**	HQ266605	XI (3g)	nd	-	-	R	-	-	*	-	V
**MG-Meola/2008**	HQ266604	XI (3g)	nd	-	-	R	-	-	*	-	V

a : ICPI intracerebral pathogenicity index;

b : Weybridge line [77];

c : [8]. The isolates from Madagascar that were subjected to analysis in this work are in bold.

### Sequencing MG, genome organisation and phylogenetic analyses

The sequence covering the six open reading frames (ORFs: NP, P, M, F, HN, L), without the complete sequence of the 3′-leader/5′-trailer of two MG strains, MG-725/08 and MG-1992, and the complete nucleotide sequence covering the F and HN genes of two other strains, MG-39-04/08 and MG-Meola/08, obtained in this work were analysed along with those of reference strains retrieved from GenBank.

**Figure 1:**
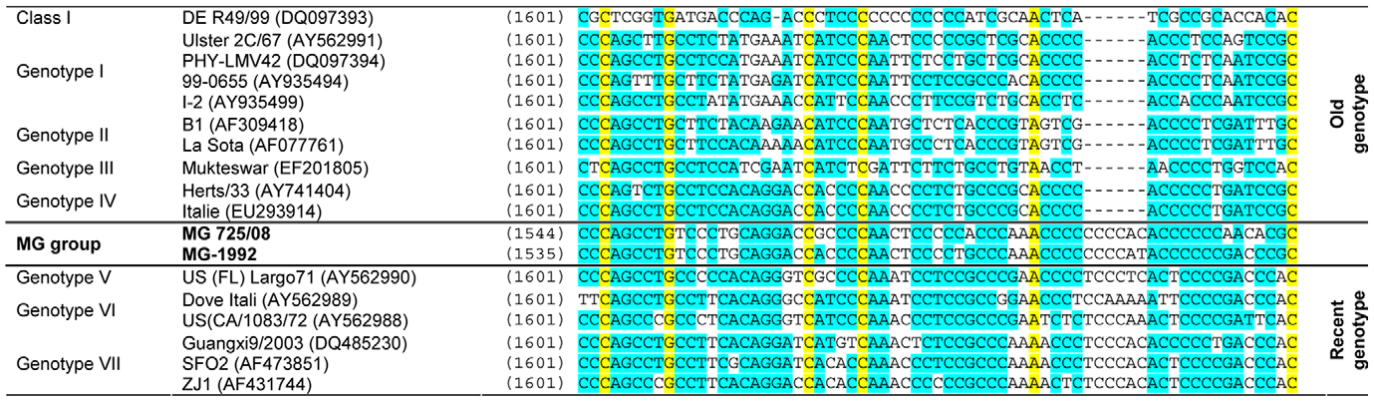
Alignment showing the inserted sequence at in the position 1647 in 5′ non-coding region of NP gene.

Class II APMV-1 isolates have two genome lengths. The genotypes considered as “early” (1930–1960; genotypes I to IV, IX and H33(W)) contain 15,186 nt and recent genotypes (that emerged after 1960, genotypes V–VIII and X) contain 15,192 nt (15,186 nt+6 nt in the 5′ non-coding region or NCR of NP gene) [12]. As seen in recent genotypes, the MG group has six nucleotides insertion in the 5′ non-coding region (NCR) of the nucleoprotein gene between nucleotides 1647 and 1648 increasing its genome size to 15,192 nt (Figure 1).

**Figure 2:**
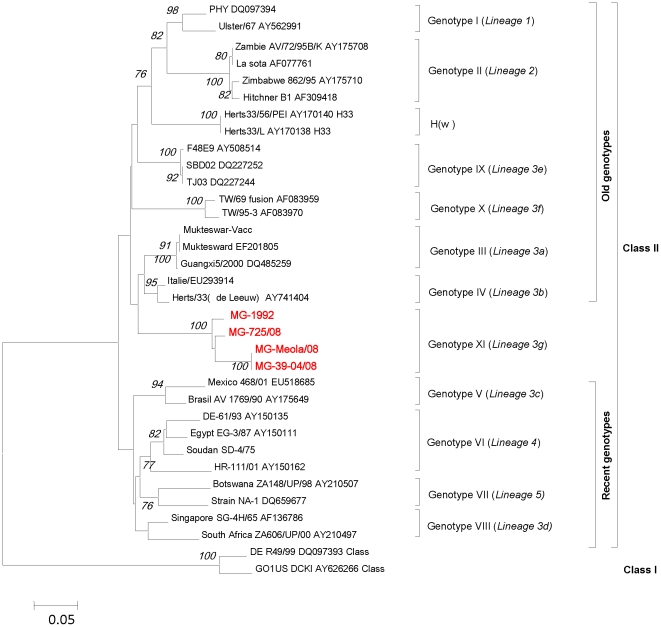
Phylogenetic tree (unrooted) of nucleotide sequences based on a 374-nt sequence (position 47–421 nt) of the F gene. Phylogenetic relationships of MG group strains with previously published sequences in Genbank. The evolutionary history was inferred using the Neighbor-Joining method [33]. All results are based on the pairwise analysis. Analyses were conducted using the Kimura 2-parameter method in MEGA4 [31], [32] with 1,000 bootstraps [76]. The isolates from Madagascar that were subjected to analysis in this work are in bold and red. Genotype or lineage groupings are indicated on the right.

**Figure 3:**
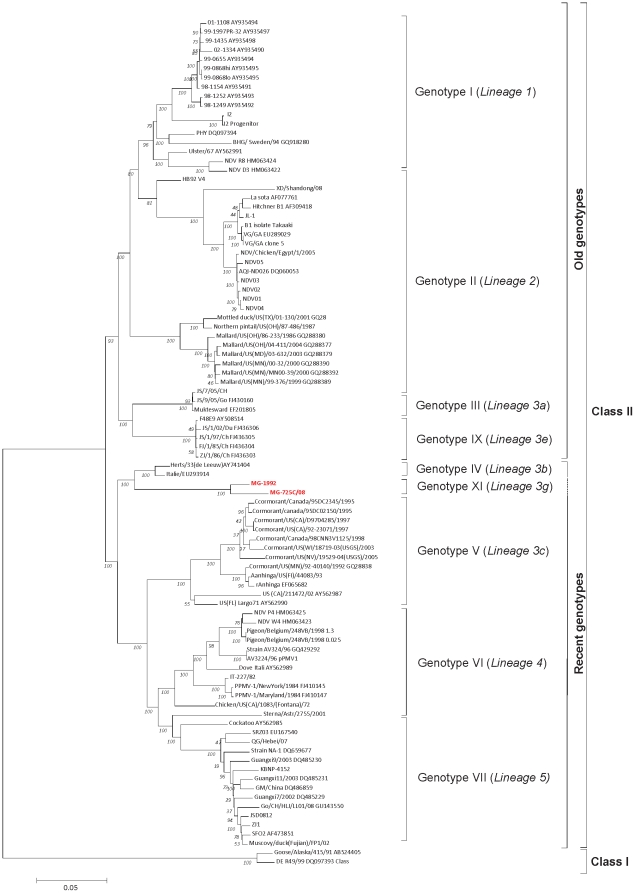
Phylogenetic tree of the nucleotide sequences based on a 14977nt (NP/P/M/F/HN/L genes). The evolutionary history was inferred using the Neighbor-Joining method [33]. All results are based on the pairwise analysis. Analyses were conducted using the Kimura 2-parameter method in MEGA4 [31], [32] with 1,000 bootstraps [76]. The isolates from Madagascar that were subjected to analysis in this work are in blue [22]. Genotype and the lineage groupings are indicated on the right.

The phylogenetic analysis of F gene (Figure 2) shows that the MG strains are grouped together. They are closer to the “old” genotype IV but may be distant enough to constitute a new genotype, proposed here as genotype XI or lineage 3g. This phylogenetic topology created using 374 nt fusion gene fragments was confirmed by phylogenetic analyses based on the full NH gene (data not shown) and on the full-genome sequences of two isolates of the MG group (MG-1992 and MG-725/08) and 86 other full-genome sequences belonging to different genotypes (Figure 3).

Based on a partial fragment of F gene (374nt), genetic divergences between the MG group and the other ten genotypes (I to X) are presented in Table 3. The F nucleotide sequences divergence within the MG group was 5%. The maximum intra-genotype divergence in old genotypes was 7% (genotypes I and IV) whereas the maximum intra-genotype divergence in recent genotypes was 9% (genotype VIII). The lower genetic divergence found between the MG group and another genotype (genotype IV) was 12%. In contrast, the higher divergence (25%) was observed with the genotype II that includes the La Sota vaccine strain. Since the maximal intra-genotype divergence ever observed does not exceed the minimal divergence of the MG group with other genotypes, our data support the hypothesis that MG group constitutes a distinct new genotype. This new genotype is further confirmed by the phylogenetic analysis of the full-genome sequences (Figure 3). Surprisingly, the phylogenetic analysis of whole genome sequences also show that the genotype IV does not cluster with the “old genotypes” (I, II, III, IX), as suggested before (Czegledi et al., 2006), but rather with “recent genotypes” (V,VI,VII).

**Table 3:** Matrix estimate of genetic divergence (%) between nucleotide sequences (374 nt) of NDV strains.

*Genotypes*	XI	I	II	H(w)	III	IV	V	VI	VII	VIII	IX	X	CI***
**XI**	**5** *												
**I**	20**	**7**											
**II**	25	12	**2**										
**H(w)**	21	15	16	**0**									
**III**	15	12	17	15	**1**								
**IV**	12	12	17	15	6	**7**							
**V**	19	16	19	18	14	11	**5**						
**VI**	18	16	19	18	14	11	11	**7**					
**VII**	21	18	21	21	17	14	14	3	**7**				
**VIII**	22	17	20	17	14	13	11	12	15	**9**			
**IX**	15	13	17	14	10	9	14	14	17	16	**1**		
**X**	15	18	19	21	15	13	19	19	19	19	15	**3**	
**CI**	50	46	51	50	48	47	47	46	47	48	49	52	**7**

*Intragenotype or intrasubgenotype: Maximal value of Nucleotide genetic divergence.

**Intergenotype: Minimal value of genetic divergence.

***Class I.

All results are based on the pairwise analysis of 61 NDV sequences including the 4 MG isolates (genotype XI). The nucleic acid analyse were conducted using Kimura 2-parameter method in MEGA4 [31], [78]. All positions containing alignment gaps and missing data were eliminated only in pairwise sequence comparisons (Pairwise deletion option). There were a total of 374 nt positions in the final dataset.

The analysis of the F_1_/F_2_ protein cleavage site sequence of the four MG isolates showed that they share an original cleavage site motif formed by five arginines (R) ^112^R-R-R-R-R^116^ at the C terminus of F_2_ exhibiting an atypical Q/R substitution at position 114 followed by a phenylalanine (↓F^117^) at position 117 of the F_1_ amino terminus (Table 2). This motif is considered as a velogenic motif according to OIE [3]. Other virulent-like cleavage motifs with five basic amino acids have been described for genotypes VI, VII and VIII that are formed by a combination of arginine (R) and lysine (K) residues like ^112^R-R-K-K-R^116^*F^117^ or ^112^R-R-R-K-R^116^*F^117^ [35], [36], [37], [38], [39]. The substitution of a glutamine residue by a basic amino acid (R or K), corresponding to nucleotide mutation at positions 4885 or 4886 (C**A**G→C**G**G or **A**AG) is only found in some strains pertaining to the “recent” genotypes VI or VIII.

Like other NDV strains, the complete length of F and HN genes of MG group viruses were 1792 nt and 2002 nt, respectively. The F gene of all MG group isolates showed a single open reading frame (ORF) beginning with a unique double start codons ^44^AUGAUG^49^ and ending at position 1705 followed by the UGA stop codon. Consequently, the open reading frame of the F gene may extend to 1665 nt rather than 1662 nt usually found in other reference strains.

The predicted amino acid sequences of the complete F gene of the four MG isolates was analysed and compared with different strains pertaining to genotypes I to IX (Table 4). This analysis showed that all seven neutralising epitopes critical for both structure and function of the protein positioned at individual residues D^72^, E^74^, A^75^, K^78^, A^79^, L^343^ and the stretch of amino acids ^157^ILRLKESIAATNEAVHEVTDG^171^, are conserved [40], [41], [42]. The twelve cysteines residues placed at positions 25, 76, 199, 338, 347, 362, 370, 394, 399, 401, 424 and 523 [43] and the predicted N-glycosylation sites (Asn-X-Ser/Thr or N-X-S/T) where X is any amino acid except aspartic acid or proline, located at positions ^85^N-R-T^87^, ^191^N-K-T^193^, ^366^N-T-S^368^, ^447^N-I-S^449^, ^471^N-N-S^473^ and ^541^N-N-T^543^ [44], [45] are also conserved in the MG group. However, the analysis of predicted amino acid sequence of a F protein fragment (residues 0–553 for MG isolates or 1–553 for the other strains) revealed substitutions in the MG isolates found also in other strains clustered in the old genotypes (I–IV and IX) like T^16^→I, E^104^→G, Q^195^→R as well as substitutions found in recent genotypes (V to VIII) like V^81^→L, A^106^→V, V^118^→I and V^350^→I. In addition, the MG group has fifteen specific amino acid residues in F protein which are not found in any other genotype (Table 4). Twelve (80%) of these amino acids are located in the head of trimeric NDV-F protein M^0^→-, W^8^→R, P^15^→L, S^272^→N, M^289^→L, S^311^→T, R^364^→S, T^371^→M, L^384^→M, I^397^→T, T^409^→S and H^411^→N. The three remaining substitutions S^244^→G, K^476^→N, and I^522^→A are located in the stalk of the F-trimeric structure (Figure 3). Interestingly, the three MG strains recently isolated in 2008 share eight additional amino acids (Q^4^→K, F^12^→P, S^28^→L, R^73^→K, S^79^→A, E^110^→G, V^202^→I, S^258^→N and H^337^→Y) compared with the strain isolated in 1992.

**Table 4:** Residue substitutions specific in deduced F_0_ protein and HN protein sequence of the MG group.

		*Consensus residue and its position*																								
		*In deduced F_0_ protein sequence*													*in deduced HN protein sequence*											
		0	8	15	244	271	311	364	371	384	397	409	411	522	24	34	39	62	78	81	98	147	263	328	430	539
		-	R	L	G	M	T	S	M	M	T	S	N	A	V	V	I	V	Q	V	N	D	N	T	T	K
**MG-1992**		M	W	P	S	T	S	R	T	L	I	T	H	I	A	T	L	E	R	M	S	E	R	I	I	R
**MG-Meola/08**		M	W	P	S	T	S	R	T	L	I	T	H	I	A	T	L	E	R	M	S	E	R	I	I	R
**MG-725/08**		M	W	P	S	T	S	R	T	L	I	T	H	I	A	T	L	E	R	M	S	E	R	I	I	R
**MG-39-04/08**		M	W	P	S	T	S	R	T	L	I	T	H	I	A	T	L	E	R	M	S	E	R	I	I	R
*Old genotype*	**I**	-	-	-	-	-	-	-	-	-	-	-	-	-	-	-	I	A	-	-	-	-	-		-	-
	**II**	-	-	-	-	-	-	-	-	-	-	-	-	-	-/I	-	-	R	-	-	-	-	-		-	-
	**III**	-	-	-	-	-	-	-	-	-	-	-	-	-	-/I	-	-	A	-	-	-	-	-/k		-	-
	**IV**	-	-	-	-		-	-	-	-	-	-	-	-	-/I	-	-	-	-	-	-	-	G/S		-	-
	**IX**	-	-	-	-	-	-	-	-	-	-	-	-	-	-	-	-	A	-	-	-	-	-		-	-
	**X**	-	-	L/Q	?	?	?	?	?	?	?	?	?	?	?	?	?	?	?	?	?	?	?		?	?
*Recent genotype*	**V**	-	-	-	-	-	-	-	-	-/I	-	-	-	-	-	-	-	-	-	-	-	-	K		-	-
	**VI**	-	-	-	-	-	-	-	-	-	-	-	-	-/V	-	-/I	-	-	-	-/I	-	-	K		-	-
	**VII**	-	-	-	-	-	-	-	-	-	-	-	-	-/G	-/I	-	-	-	-	-	-	-	K		-	-
	**VIII**	-	-	-	-	-	-	-	-	-	-	-	-	-	?	?	?	?	?	?	?	?	?	?	?	?

? : Sequence not available.

**Figure 4:**
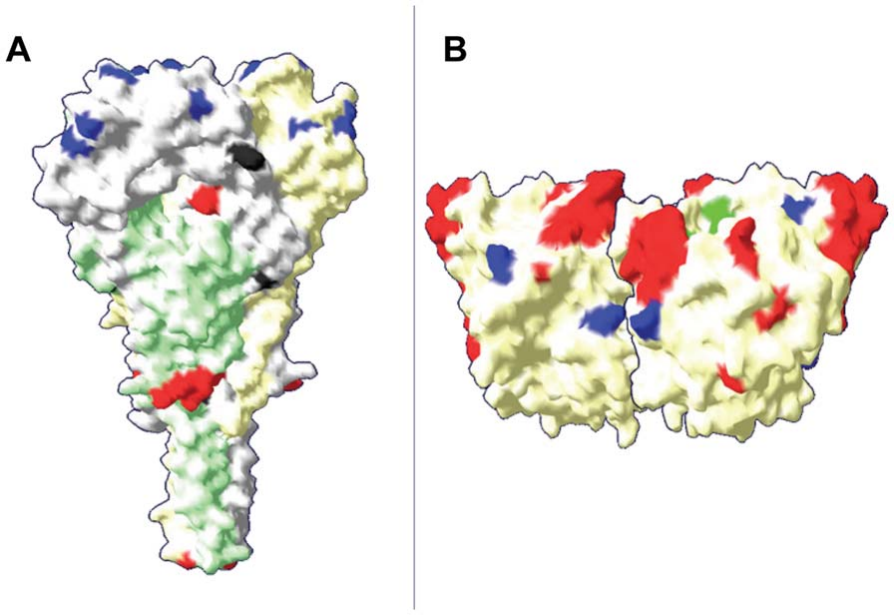
F-NDV trimer (a) and HN-NDV dimer (b) surface representations of the MG strains. The location of neutralising epitopes is shown in red, the amino acid substitutions specific to the MG group are in blue in the globular head and in black in the stalk region. The figures were generated by Swiss PDB-viewer, using the X-ray structure of NDV F protein, position 33aa to 454aa for F protein [45] and 124aa to 569aa for HN protein [51].

According to the virulence of NDV strains, HN monomeric protein sequences possess different amino acid sequence lengths: 571, 577, 581 and 616 [46]. The ORF of HN gene of MG group strains begins at position 92 and ends at position 1714. This HN protein is composed of 571 aa and has the feature of virulent NDV strains [14], [42], [46], [47]. In this study, different HN sequence strains pertaining to genotypes or subgenotypes available in Genbank (I to VII and IX) with different lengths were aligned with the MG group sequences (data not shown). The results showed that all the previously described neutralising epitopes ^193^LSGCRDHSH^201^, R^263^, D^287^, K^321^, ^332^GR^333^, ^346^DEQDYQIR^353^, K^356^, N^481^, D^494^, ^513^RITRVSSSS^521^, G/D^569^ [48], [49], [50] are not modified in MG group strains. The three amino acid E^401^, R^416^, Y^526^ essential for receptor binding site [51], [52] and the neuraminidase activity site represented by functional triarginyl cluster at positions ^174^RI^175^, R^416^, R^498^, the amino acid sequence in region ^234^NRKSCSI/V/L^240^ [53] as well as the regions ^314^FXXYGGV/L/M^320^ - ^399^GA/SEGRI/V/L^405^ involved in hemagglutinating activity [54], were likewise conserved in the MG strains. In addition, the MG strains have preserved the thirteen cysteines residues in the linear sequence of the ectodomain 123, 172, 186, 196, 238, 247, 251, 344, 455, 461, 465, 531, 542 [43] and the eleven sialic acid receptor binding sites R^174^, I^175^, E^258^, Y^299^, Y^317^, E^401^, R^416^, R^498^, Y^526^, R^516^, E^547^ [52]. Except the cysteine residue at position 123, all these amino acid cited before are located in the globular head on the HN spike. Otherwise, the analysis of HN amino acid sequences showed that the four MG strains contain also eleven specific amino acid residues that are different from those of any other NDV strain (Table 4). Seven of these A^24^→V, T^34^→V, L^39^→I, E→V^62^, R→Q^78^, M^81^→V and S^98^→N are located in the 124 first N-terminal amino acids, including the cytomere (position 1 to 20), the transmembrane region (position 21 to 49) and the stalk (position 50 to 124) of HN protein. The four last characteristic amino acids E^147^→D, R^263^→N, I^328^→T, R^539^→K are localized on the globular head of the protein (Figure 4). In addition, the MG-725/08 strain has six additional specific substitutions H^3^→R, T^64^→S, V^117^→A, R^377^→K, A^380^→T and A^510^→S when compared with the three other MG strains (Meola/08 MG 39-04/08 and MG-1992).

The analysis of recombination events by the RDP 3.0b software [34] was performed among Malagasy strains and other full sequences of APMV-1 of class II (genotypes I, II, III, IV, V, VI, VII and XI) and one strain of class I. No recombination events were detected in the Malagasy strains.

## Discussion

In this study, complete genome or complete genes of F and HN proteins of four NDV strains isolated in Madagascar (MG group strains) were sequenced and analysed. These strains are the first Madagascar isolates characterised at the molecular level. Among them, the strains MG 1992 and MG39-04/08, isolated in 1992 and 2008 respectively, were recovered from vaccinated chickens.

The salient findings of this work are the strong evidence that MG isolates constitute a new genotype closer to the old genotypes that were circulating 50 years ago and responsible for the first pandemic of ND in the world. The MG group strains are branched to the genotype IV cluster from which strains have never been detected since 2002. [12]. The partial sequence of the F gene (374 nt at positions 47 to 420) is regarded as a standard criterion for NDV genotyping of isolates present in various parts of the world [55]. Based on percentage of genetic divergence of this F gene fragment, the maximum intra-genotype nucleotide variations within any old genotype (below 7%) is lower than the minimum nucleotide distance found between these genotypes and the MG group (12% to 25%). This is clearly supporting that MG isolates are closely related to genotype IV but distant enough to constitute a new genotype or lineage, named here genotype XI or lineage 3g in reference to the two last genotypes IX [42] and X [14] (lineages 3e and 3f, respectively) identified in Asia. The cluster topology of the MG group strains based on the F gene was confirmed by the phylogenetic analysis based on full-genome sequences of two isolates of the MG group (MG-1992 and MG-725/08) and 86 other full-genome sequences belonging to different genotypes.

Whole genome sequencing of two MG strains revealed a genome size of 15,192 nucleotides meaning that this group has an insertion of six nucleotides into the 5′ NCR of the nucleoprotein gene, characteristic of recent genotype strains. In addition, MG isolates have an arginine at position 114 (R^114^) of the F protein cleavage site found in some recent isolates but never in old strains. MG viruses have also a substitution V^118^→I, which is considered to be a feature of the genotype V [56], [57] that belongs to the recent genotypes cluster. In contrast, MG isolates share a molecular substitution characteristic of old genotypes as E^104^→G, in F protein [42], [58]. Although Yu et al [58] claimed that E^104^→G substitution caused a dramatic change responsible for the evolution of the old genotypes towards the recent genotypes, we do not found such mutation in the MG strains nor in the strains of genotypes IX and X, phylogenetically closer to the old genotypes. Moreover, the phylogenetic analysis based on full-genome sequences unexpectedly showed that the genotype IV, and the newly proposed genotype XI as well, are not branched in the “old genotypes” cluster (I, II, III, IX), as observed in phylogenetic trees constructed based on the 374 nt fragment of F gene. All together, our results can suggest that genotypes IV and XI are intermediates between old and new genotypes or that the current system of genotypes or lineages clustering based on the 374 nt fragment of F gene is probably inadequate to classify NDV isolates.

All MG isolates show the same fusion protein cleavage motif ^112^R-R-R-R-R*F^117^. To our knowledge, this atypical virulent-like cleavage site has never been reported before in APMV-1 strains. However, it is found in other paramyxoviruses (*Rubulavirus*) like the simian virus type 5 [45], [59]. In addition, the presence of a phenylalanine at position 117 (↓F^117^) in the MG isolates was previously described as being a possible contributor to neurological effects [10]. Thereafter, the length of amino acid sequence of the haemagglutinin-neuraminidase protein (571 aa) is also characteristic of virulent strains [46], [60], [61]. The prediction of virulence based on the F cleavage site pattern was confirmed by *in vivo* tests that has resulted in a high ICPI value (1.9), close to the maximum of 2, with the two strains tested (MG-1992 and MG-725/08). In spite of the established virulence of the MG-725/08 strain, it was recovered both from cloacal and tracheal swabs from an unvaccinated and apparently healthy chicken. However, the possibility that this chicken was sampled during the incubation period of the infection or was partially protected by a previous infection with an avirulent or vaccine strain that circulate in the field cannot be ruled out.

Based on neutralising tests and cross-protective analyses, it is accepted that APMV-1 exists as a single serotype [62]. Therefore, the genetic variations of the virus are not expected to result in vaccination failure. However, different levels of cross-protection have been observed in chickens vaccinated with the vaccine strain La Sota belonging to the genotype II and challenged with different wild-type strains [55]. Sporadic cases of ND in commercial farms vaccinated with this vaccine were previously reported in China [7], [58], in southern California and adjacent states [63], in Mali [64], Cameroon, Nigeria and Burkina Faso [65]. Whether these observations are related to a reduction of vaccine efficacy or an improper vaccine use in the field remains unclear. It is however possible that NDV strains responsible for ND sporadic outbreaks in vaccinated chickens can escape the immune responses [16], [18], [66] and thus contribute to the emergence of new genotypes [42]. This vaccine failure has been announced by Alexander et al., [67] during the third panzootic of ND (1981–1983) in Europe in which the pigeons immunized with live NDV B1 vaccine were not well protected against pigeon paramyxovirus isolates (genotype VI). It is also known that the immune pressure imposed by the vaccination may be selecting virulent variant forms of NDV [68]. Many authors have demonstrated that current vaccines prevent disease but cannot stop viral shedding [47], [63]. The NDV strains currently circulating (subgenotype VIId prevalent in Asia, Europe and Africa) and the genotype XI predominant in Madagascar have a significant genetic distance (21% to 25%, respectively), with the widely used vaccine strain La Sota pertaining to the genotype II. These genetic differences and a consequent suboptimal vaccination may be responsible for sporadic cases of ND in vaccinated poultry flocks in Madagascar. In contradiction with this, multiple sequence alignments of the four MG isolates made with several published sequences including the La Sota strains and HB1, members of genotype II (data not shown) revealed that all the neutralising epitopes identified so far and the predicted attachment receptor sites in the F and HN glycoproteins are conserved. However, all MG group strains possess fifteen and eleven original amino acid substitutions on F and HN proteins, respectively. Some of these substitutions occurred in the globular head of the proteins. Furthermore, 5 out of the 8 mutations in the head of the F protein are concentrated in a short invariable region at position 364–411, suggesting that the observed mutations are actually selected over time by the host immune responses. It is supposed that these substitutions should have resulted in antigenic drift and major changes in the protein conformation [69], particularly the changes in amino acid polarity as T^34^→V, E^62^→V in HN protein and S^244^→G in F protein. The HN/F protein interaction for fusion promotion involves the HN head region (aa 124 to 151) and the F protein heptad repeat 2 (HR2) at position 454 to 492 [70], [71]. In these regions, the MG strains show amino acids substitutions at position E^147^→D, K^476^→N of HN and F proteins, respectively. Morrison and Gravel [72] have demonstrated that amino acid substitutions in the head region domain of HN protein as L^133^→I or A^140^→L were responsible for an enhanced or diminished virus attachment activity, respectively. The possibility that other mutations like E^147^→D e/or K^476^→N in the same or related functional region may also contribute to virulence or immune evasion cannot be ruled out. In addition, the MG isolates display other specific mutations, some of them affecting the head of HN. It is tempting to postulate that these modifications (F or HN genes) may play a role in virulence or emergence of escape mutants and finally the apparent lack of vaccine efficacy observed in the field.

The number of unique motifs found in all MG isolates, including the atypical F protein cleavage site R-R-R-R-R*F-V, also suggests a monophyletic origin of these viruses. Under this hypothesis, the maximum genetic variation observed within MG isolates (5%) is compatible with a divergence from a common ancestor over 50 years, considering that the rate of nucleotide change is approximately 1% per decade under natural field conditions in epizootic periods [12], [57]. This scenario is in agreement with the first introduction of an old genotype of NDV in Madagascar in 1946. However, the higher genetic variation (12%) observed between the genotype XI and its closely related genotype IV suggests that the common ancestor of these two genotypes may have emerged several decades before the introduction of its virus progeny in Madagascar. As mentioned before, the accumulation of multiple aa substitutions in F or HN proteins may also result from the immune pressure that can contribute to increase the phylogenetic distance between the MG group and its progenitor genotype. The presence of this selection pressure at specific amino acid sites are recognised as adaptive evolution [47], [73]. This abnormal evolution rate of genotype XI with regard to genotype IV may originate from combined characteristics of current poultry production systems including the commercial farm with host genetic homogeneity, intensive and/or improperly executed vaccination programs and backyard poultry breeding with high density of birds (allowing close animal-to-animal contact, and favouring transmission of highly virulent virus over milder forms) [74]. Moreover, high phylogenetic and antigenic distances between vaccines and circulating strains may facilitate the evolution of virulent NDV [75].

In conclusion, all four isolates from Madagascar were clustered in a new genotype XI, presumably deriving from an ancestor close to genotype IV introduced in the 50's and possibly resulting from a self-contained evolution due to geographical and ecological characteristics of this island. The particular evolution of genotype XI in Madagascar reinforces the idea that this island, known for its outstanding flora and fauna biodiversity, is also a unique “natural ecosystem” for micro-organisms. Other genotypes like genotypes I, II, III and VII were also detected in the island (data not shown). These genotypes may have been introduced more recently in the island, probably as a consequence of poultry import or use of live vaccine strains. The co-circulation of different genotypes at the same time and other introductions of new viruses in relation with the worldwide intensification of animal movements, may contribute in the future to dramatically increase the complexity of the situation.

## Supporting Information

Table S1 Primers used in this study.
(0.07 MB DOC)

Table S2 Accession numbers of the sequences used in this study.
(0.12 MB DOC)
