# Supplementary material for: Newcastle Disease Virus in Madagascar: Identification of an Original Genotype Possibly Deriving from a Died Out Ancestor of Genotype IV
Source: PLoS One. 2010 Nov 15;5(11):e13987. doi: 10.1371/journal.pone.0013987 (PMC2981552; doi:10.1371/journal.pone.0013987)
Supplement: Table S1 — Primers used in this study. (0.07 MB DOC) [file pone.0013987.s001.doc]

**Table S1. Primers used in this study.**

| **Name primers** | **Sequence 5'-3'** | **PCR length** | **Reference** |
| --- | --- | --- | --- |
| 1U | AAGGCGAAGRAGCAATCGAA | 520 | - |
| 1L | GGGTTCCGTTRCTGCATGC |
| 2U | CCCCAGTTCAACAAYAGGAG | 637 | - |
| 2L | GGTGCAAAGCTCATCTGGTC |
| 3U | AAAAAATGAARCAGCTCATGCG | 599 | - |
| 3L | TCGRTCAGTACCCCCAGTC |
| 4U | AGCGGTGGCAAAYAGCATG | 587 | - |
| 4L | GGAYGCTCCCATGCTTCTC |
| 5U | TTGGAAGGAGYGCAATCCC | 692 | - |
| 5L | GCTGATAGTCAACTTTAYTTAC |
| 6U | GCACCTGTGGATCATGTCCA | 719 | - |
| 6L | GATGCAGCCGGRTCGATTG |
| 7U | AGARAGACACTGTCCGTGC | 721 | - |
| 7L | TRTTAGTTGCACTCTTCTTGCA |
| 8U | CRATGCTCTGCCTAGGRAG | 564 | - |
| 8L | CACGGAAGGTCCGAGCAC |
| 9U | GAAGAAAGTGACATTTGACAAG | 624 | - |
| 9L | AGCTGCAAGAGGCCTGCC |
| 10U | TAGAAAAAACACGGGTAGAAGA | 594 | - |
| 10L | CCAACTGCCACTGCTAGTTG |
| MFS1 | GACCGCTGACCACGAGGTTA | 699 | (Aldous *et al.*, 2004) |
| MFS2 | GCTGCCAACATCCTCCGACT | (4306-5005) | - |
| F+4952 | GCAGCCGCAGCTCTAATAC | 1534 | (Aldous *et al.*, 2004) |
| #33 | AGTAAGATTGCGATCCGG | (4952-6486) | - |
| P6A | ATCAGATGAGAGCCACTACA | 1165 | (Zou *et al.*, 2005) |
| P6Bm | TAGACTGGGAACCATACGC | (6177-7342) | - |
| P7A | GGGTTTGACGGCCAATACCA | 1153 | (Zou *et al.*, 2005) |
| P7B | TCTGCCCTTTCAGGACCGGA | (7237-8390) |
| 17U | CATGTTTTAAAGTTGTCAAGAC | 562 | - |
| 17L | GATTCAAGTATTTTYTTCCATTG |
| 18U | ACAAACTACTCTAYTACTGGAA | 645 | - |
| 18L | TCCATCATATCYGCATACATC |
| 19U | TTGTTACTCCTGAGCTTGTC | 591 | - |
| 19L | TTCTTTCTGTATCCRTTGATGA |
| 20U | GAGCCARATGTGCGCACC | 543 | - |
| 20L | CTTAGCRAAAATCCGCCCATT |
| 21U | TTGAGTACCTAAGAGATGACA | 634 | - |
| 21L | TCTTGCTGCAGCAAGTTGGA |
| 22U | GATGAYATATATATTGTCAGTGC | 597 | - |
| 22L | CAAAAGAGATGTCYTCAATCCA |
| 23U | ACTTAAACTACCTAATGAGTTG | 639 | - |
| 23L | ATCCGCATCAGCCTCTTGAT |
| 24U | AAGCAAGCTCTGTAGGTAGG | 568 | - |
| 24L | GTTGTCYCCATAAGCCCAG |
| 25U | TTGCAAAAATAGCYCATATGTC | 590 | - |
| 25L | GCTCATAACTCTTRAAGATAGC |
| 26U | AATAAGTTTATGTATGATCCTAG | 514 | - |
| 26L | GGAACAGCAGATCRTACTTATT |
| 27U | CGGATTTTATYGAAATGTCTGC | 626 | - |
| 27L | CTCTGAACARGTAYCGTACTA |
| 28U | GATTTGAGTGCYCCAGCAAG | 603 | - |
| 28L | CTTCCCTTACAGARTGCATG |
| 29U | GACATTGARATTCCTCCAGG | 566 | - |
| 29L | TCGAATGACTGTGTCAATGTG |
| 30U | GCCCGTCCGTCCATTCTG | 698 | - |
| 30L | TACCAAACAAAGATTTGGTGAA |
