# Supplementary material for: Newcastle Disease Virus in Madagascar: Identification of an Original Genotype Possibly Deriving from a Died Out Ancestor of Genotype IV
Source: PLoS One. 2010 Nov 15;5(11):e13987. doi: 10.1371/journal.pone.0013987 (PMC2981552; doi:10.1371/journal.pone.0013987)
Supplement: Table S2 — Accession numbers of the sequences used in this study. (0.12 MB DOC) [file pone.0013987.s002.doc]

Table S2. Accession numbers of the sequences used in this study.

| **Strain** | |
| --- | --- |
| **Identification** | **Accession number** |
| DE R49/99, | DQ097393 |
| Goose/Alaska/415/91 | AB524405 |
| Ulster/67 | AY562991 |
| PHY-LMV42/66 | DQ097394 |
| Hitchner B1 | AF309418 |
| La Sota | AF077761 |
| Mukteswar | EF201805 |
| Herts/33 (de Leeuw) | AY741404 |
| 99-0655 | AY935494 |
| 99-0868hi | AY935495 |
| 99-0868lo | AY935495 |
| 01-1108 | AY935494 |
| 99-1435 | AY935498 |
| 99-1997PR-32 | AY935497 |
| 02-1334 | AY935490 |
| 98-1154_ | AY935491 |
| 98-1252 | AY935493 |
| 98-1249 | AY935492 |
| I-2 | AY935499 |
| I2_Progenitor | AY935500 |
| NDV_R8 | HM063424 |
| NDV_D3 | HM063422 |
| BHG/_Sweden/94 | GQ918280 |
| Mallard/US(OH)/86-233/1986 | GQ288380 |
| Mottled_duck/US(TX)/01-130/2001 | GQ288391 |
| Mallard/US(OH)/04-411/2004 | GQ288377 |
| Mallard/US(MN)/MN00-39/2000 | GQ288392 |
| Mallard/US(MD)/03-632/2003 | GQ288379 |
| Mallard/US(MN)/99-376/1999 | GQ288389 |
| Mallard/US(MN)/00-32/2000 | GQ288390 |
| strain_VG/GA_ | EU289029 |
| AQI-ND026_ | DQ060053 |
| Northern_pintail/US(OH)/87-486/1987 | FJ705473 |
| VG/GA_clone_5 | EU289029 |
| B1_isolate_Takaaki | AF375823 |
| XD/Shandong/08 | GQ994433 |
| NDV/Chicken/Egypt/1/2005 | FJ939313 |
| HB92_V4 | AY225110 |
| JS/7/05/CH | FJ430159 |
| Muktesward_ | EF201805 |
| JS/9/05/Go_ | FJ430160 |
| Italie | EU293914 |
| cormorant/Canada/95DC2345/1995 | GQ288384 |
| cormorant/US(CA)/D9704285/1997 | GQ288381 |
| cormorant/Canada/98CNN3V1125/1998 | GQ288382 |
| cormorant/US(WI/18719-03(USGS)/2003 | GQ288385 |
| cormorant/US(NV)/19529-04(USGS)/2005 | GQ288386 |
| cormorant/US(CA)/92-23071/1997 | GQ288388 |
| cormorant/canada/95DC02150/1995 | GQ288383 |
| Cormorant/US(MN)/92-40140/1992 | GQ28838 |
| F48E9 | AY508514 |
| JS/1/97/Ch | FJ436305 |
| JS/1/02/Du | FJ436306 |
| FJ/1/85/Ch | FJ436304 |
| ZJ/1/86/Ch_ | FJ436303 |
| NDV_P4 | HM063425 |
| anhinga/US(FI)/44083/93 | AY562986 |
| US_(CA)/211472/02 | AY562987 |
| US(FL)_Largo71 | AY562990 |
| rAnhinga | EF065682 |
| Dove_Itali_ | AY562989 |
| strain_AV324/96 | GQ429292 |
| AV3224/96_pPMV1 | GQ429292 |
| IT-227/82 | AJ880277 |
| PPMV-1/NewYork/1984 | FJ410145 |
| PPMV-1/Maryland/1984 | FJ410147 |
| pigeon/Belgium/248VB/1998_1.3 | EF026583 |
| pigeon/Belgium/248VB/1998_0.025 | EF026579 |
| chicken/US(CA)/1083/(Fontana)/72 | AY562988 |
| Strain_NA-1 | DQ659677 |
| SFO2 | AF473851 |
| Cockatoo | AY562985 |
| Guangxi9/2003 | DQ485230 |
| Guangxi11/2003 | DQ485231 |
| Guangxi7/2002 | DQ485229 |
| SRZ03 | EU167540 |
| GM/China | DQ486859 |
| Go/CH/HLJ/LL01/08 | GU143550 |
| ZJ1 | AF431744 |
| JSD0812 | GQ849007 |
| KBNP-4152 | DQ839397 |
| Muscovy/duck(Fujian)/FP1/02 | FJ872531 |
| QG/Hebei/07 | GQ994434 |
| Sterna/Astr/2755/2001 | AY865652 |
| NDV01 | FJ386392 |
| NDV02 | FJ386393 |
| NDV03 | FJ386394 |
| NDV04 | FJ386395 |
| NDV05 | FJ386396 |
| JL-1 | EU546165 |
| DE R49/99 | DQ097393 |
| GO1US DCKI | AY626266 |
| Herts33/56 (PEI) | AY170140 |
| Herts33 (L) | AY170138 |
| Ulster/67 | AY562991 |
| Ethiopie Panvac (2/P2) | AY175720 |
| PHY-LMV42/66 | DQ097394 |
| Hitchner B1 | AF309418 |
| La Sota | AF077761 |
| Zimbabwe AV862/95 | AY175710 |
| Zambie AV 72/95 | AY175708 |
| Mukteswar | EF201805 |
| Guangxi5/2000 | DQ485259 |
| Herts/33 (de Leeuw) | AY741404 |
| BG 60-81 | AF402129 |
| BG 5-67 | AF402104 |
| SIMF/64 | AJ243390 |
| Soudan 72 AV 2203 | AY135753 |
| DE-191/77 | AF525378 |
| IT-48/68 | AF297969 |
| MA-307/77c | EU604259 |
| MA-13/02 c | DQ096598 |
| Tanzania AV 1300/95 | AY175687 |
| Mexico468/01 | EU518685 |
| Brasil AV1769/90 | AY175649 |
| HR-111/01 | AY150162 |
| Soudan SD-4/75 | AY151384 |
| Egypte EG-3/87 | AY150111 |
| DE 61/93 | AY150135 |
| Strain NA | DQ659677 |
| MZ 13/94 | AF136775 |
| Botswana ZA148/UP/98 | AY210507 |
| South Africa ZA606/UP/00 | AY210497 |
| Singapore SG-4H/65 | AF136786 |
| F48E9 | AY508514 |
| SBD02 | DQ227252 |
| TJ03 | DQ227244 |
| TW/69 | AF083959 |
| TW/95-3 | AF083970 |
